# Subcutaneous Immunotherapy with Mannan-Conjugated Birch Pollen Allergoids in a Pre- and Co-Seasonal Treatment Regimen: An Exploratory Post Hoc Subgroup Analysis of Safety and Tolerability

**DOI:** 10.3390/jcm15145532

**Published:** 2026-07-15

**Authors:** Esther Raskopf, Gregor Pollok, Ludger Klimek, Oliver Pfaar, Christian Neuhof, Anna Rybachuk, Nadine Katzke, Hacer Sahin, Silke Allekotte, José Luis Subiza, Miguel Casanovas, Mandy Cuevas, Laura Day, Sandra del Pozo

**Affiliations:** 1 ClinCompetence Cologne GmbH, 50668 Cologne, Germany; 2 Institute of Medical Statistics and Computational Biology, Faculty of Medicine, University of Cologne, 50937 Cologne, Germany; 3 Center for Rhinology & Allergology, 65183 Wiesbaden, Germany; 4 Department of Otorhinolaryngology, Head and Neck Surgery, Section of Rhinology and Allergy, University Hospital Marburg, Philipps-Universität Marburg, 35043 Marburg, Germany; 5 Inmunotek S.L., Alcalá de Henares, 28805 Madrid, Spainsdelpozo@inmunotek.com (S.d.P.); 6 Department of Otorhinolaryngology, Head and Neck Surgery, Faculty of Medicine (and University Hospital) Carl Gustav Carus, TUD Dresden University of Technology, 01069 Dresden, Germany

**Keywords:** birch pollen allergy, allergen immunotherapy, subcutaneous immunotherapy, safety, tolerability, allergoid, mannan

## Abstract

**Background/Objectives:** Previous studies have demonstrated the safety of pre-seasonal treatment with the mannan-conjugated birch pollen allergoid EP-088-T502. However, the safety of a combined pre- and co-seasonal treatment regimen has not yet been investigated. As climate change is associated with earlier and less predictable onset of birch pollen seasons, planned pre-seasonal allergen immunotherapy may unintentionally overlap with natural pollen exposure. Therefore, evaluation of the safety of treatment administered during the pollen season is of increasing clinical relevance. This study aimed to compare, in a purely descriptive manner, the safety and tolerability of pre-seasonal versus pre- and co-seasonal treatment with EP-088-T502. **Methods:** In this prospective, open-label, phase III trial (T502-SIT-059) (EudraCT No.: 2022-004082-20), patients (N = 109) who had participated in a preceding pivotal phase III study were offered continuation treatment with active EP-088-T502 (10,000 mTU/mL) across five treatment visits. For the subgroup analysis, all patients who completed their last treatment visit before 9 April 2023 (and, thus, before the start of the birch pollen season in Germany) were assigned to the pre-seasonal group (N = 20). Those who performed the last treatment visit thereafter were assigned to the pre-/co-seasonal group (N = 83). Due to post hoc subgroup allocation and unequal subgroup sizes, all subgroup analyses were purely descriptive. **Results:** No deaths nor serious adverse events (SAEs) were reported during the study. No epinephrine administration was required. Systemic adverse drug reactions (SADRs, N = 3) occurred in two patients who had previously received placebo. No grade III or IV systemic reactions, according to the German AWMF classification, were observed. Patients receiving pre- and co-seasonal treatment developed smaller wheals (mean diameter) compared with the pre-seasonal group (immediate reactions: 0.6 vs. 0.7 cm; late-phase reactions: 0.3 vs. 0.4 cm at the last treatment visit). This was also reflected in the medians (immediate reactions: 0.2 cm vs. 0.4 cm; late-phase reactions: 0.2 vs. 0 cm at the last treatment visit). Of all AEs that were (possibly) related to EP-088-T502 (N = 89), 74 (83%) occurred at the first three treatment visits (before the birch pollen season). The frequency of AEs appeared descriptively similar between groups for the last two treatment visits. Patients who had received placebo in the previous trial experienced more treatment-related side effects compared to patients who had already received EP-088-T502 in the previous year. **Conclusions:** These data suggest that EP-088-T502 is safe and well-tolerated, even when administered during the birch pollen season, regardless of prior exposure to EP-088-T502.

## 1. Introduction

Successful allergen immunotherapy (AIT) requires not only clinical efficacy but also a favourable safety and tolerability profile. The efficacy of AIT is closely linked to the cumulative allergen dose administered. However, a high allergen dose carries a higher risk of side effects, so the dose per application is limited. In addition, when treating seasonal allergies, such as grass or birch pollen allergies, allergen exposure resulting from AIT and natural pollen exposure may accumulate and result in increased cumulative allergen exposure, which, in turn, may increase the risk of systemic allergic reactions. Pre-seasonal AIT has proven to be safe, effective, and with few side effects [1,2]. When allergy-typical symptoms occur, co-seasonal AIT is administered either with a reduced maintenance dose or over a few days at a low initial dose until the maintenance dose is reached at the beginning of the pollen season when pollen counts are low [3].

However, tolerability of the treatment does not only depend on the time point of administration (before and/or during the pollen season), but also on the composition of the administered allergen, like native allergen extracts or (conjugated) allergoids. Native allergen extracts have been used in AIT for decades, but incidences of adverse drug reactions are high [4]. Modified allergen extracts like allergoids are a promising alternative to reduce adverse reactions to AIT. Allergoids are chemically modified allergens in which IgE epitopes are destroyed or masked by glutaraldehyde or formaldehyde, thereby reducing allergenicity whilst largely preserving T-cell reactivity [5]. The main advantage of allergoids is their reduced allergenicity while maintaining immunogenicity, resulting in an improved safety profile. The risk of IgE-mediated adverse drug reactions, such as systemic reactions and anaphylaxis, is significantly reduced during treatment [5].

The addition of adjuvants to allergoids further improves the immune response: Adjuvants increase the immune response directed against the co-administered antigen, thus enabling the administration of a lower antigen dose with fewer side effects. At the same time, they trigger an enhanced immune response against otherwise non- or low-immunogenic antigens. First-generation adjuvants include aluminium salts, calcium phosphate, and microcrystalline tyrosine = MCT, while second-generation adjuvants include TLR ligands (flagellin, CpG, and monophosphoryl lipid A = MPLA) [6]. Another adjuvant is mannan, a β-1,4-mannose polysaccharide found in the cell walls of plant and fungal cells and isolated from yeast cells. As a naturally occurring substance, mannan can, therefore, be rapidly absorbed by the body. Non-oxidised mannan is conjugated to allergoids using glutaraldehyde as a crosslinker. The mannan–antigen complex is taken up by dendritic cells via C-type lectin receptors through endocytosis and activates T helper and T regulatory cells [6].

Mannan-conjugated birch pollen allergoids have previously been investigated in several studies, including two DBPC studies and one open long-term study. In those studies, pre-seasonal treatment with the mannan-conjugated birch pollen allergoid was effective and safe. These findings are clinically relevant given the high prevalence of pollen allergy in Europe [7]. In the preceding pivotal phase III trial, treatment with EP-088-T502 significantly improved symptom and medication scores compared with placebo, and demonstrated a favourable safety profile with a low incidence of systemic adverse reactions. These findings provided the basis for the present follow-up study, evaluating safety under conditions of potential seasonal pollen exposure [1].

The treatment of patients with pollen allergy is further complicated by the fact that birch pollen seasons have been starting progressively earlier due to climate change [8,9,10]. Although the AIT was planned well in advance, planned pre-seasonal treatment may unintentionally overlap with the pollen season, as the start of the pollen season may be sooner than expected. In such cases, it is important to know whether treatment is safe during the pollen season, making it clinically relevant to evaluate the safety of treatment during ongoing pollen exposure.

The aim of this study was to compare the safety profile of a pre-seasonal SCIT with mannan-conjugated birch pollen allergoids (EP-088-T502) with the pre- and co-seasonal treatment in patients who were previously treated with either EP-088-T502 or placebo [1]. This was achieved by analysing treatment-emergent adverse events—especially adverse drug reactions like local and systemic reactions—and the use of rescue medication in both groups.

## 2. Materials and Methods

### 2.1. Study Design

This was an open-label, uncontrolled, non-randomised phase III follow-up study conducted at 13 study centres in Germany. Participants who completed the previous study T502-SIT-045 (EudraCT No. 2021-002252-36), regardless of treatment assignment in the preceding study (either EP-088-T502 or placebo), were invited to take part. For details on this preceding study, please refer to Mösges et al., 2025 [1].

The study comprised seven visits: one combined screening/first treatment visit (V1); four treatment visits at intervals of 7–14 days (V2–V5); one visit during the peak birch pollen season in mid-April 2023 (V6); and one final follow-up visit after the birch pollen season in May 2023 (V7).

**Figure 1:**
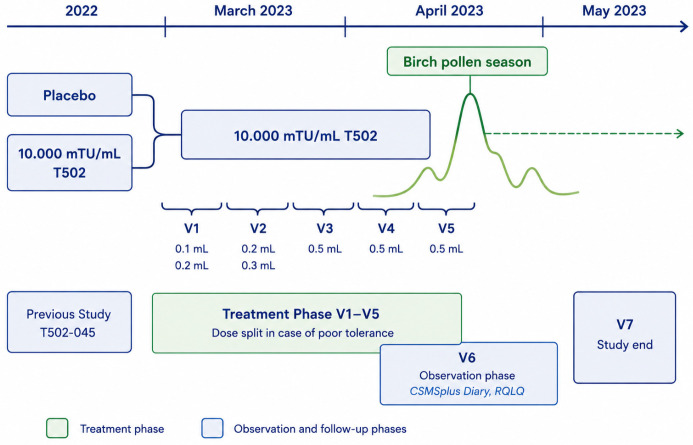
Overview of the T502-SIT-059 study.

The study procedure is shown in Figure 1.

### 2.2. Allergen Immunotherapy

Immunotherapy was performed with the injection solution EP-088-T502, a polymerised allergen extract from birch pollen allergens of *Betula pendula* conjugated to mannan using glutaraldehyde [2].

During the treatment phase, patients received five doses of EP-088-T502 (10,000 mTU/mL), injected subcutaneously into the upper arm(s), with the dose divided into two injections of 0.1 mL (1000 mTU) and 0.2 mL (2000 mTU) at V1 and 0.2 mL (2000 mTU) and 0.3 mL (3000 mTU) at V2. At V3 to V5, single injections of 0.5 mL (5000 mTU) were administered (Figure 1), resulting in a cumulative dose of 23,000 mTU.

### 2.3. Safety and Tolerability

At each visit, a physical examination was performed, vital signs were checked, and, in asthmatics, lung function was tested before and after treatment with EP-088-T502.

Wheals and redness at the injection site were measured by investigators and reported as local reactions (LRs) 30 min after each injection. In addition, LRs and other AEs (e.g., systemic reactions (SRs)) were documented by the patients in the evening of the injection day and during two subsequent days using a treatment diary. LRs were categorised as immediate (within 30 min) or late-phase (>8 h after injection) of the EP-088-T502. Classification of wheals into severity was based on mean wheal diameter (immediate reaction: mild: <5 cm, moderate: 5–10 cm, severe: >10 cm; late-phase reaction: mild: <10 cm, moderate: 10–20 cm, severe: >20 cm).

At V1, patients were provided with 10 tablets of bilastine (20 mg) for as-needed treatment of adverse drug reactions (ADRs), in accordance with the Summary of Product Characteristics. Intake of rescue medication was recorded by the patients in the treatment diary for up to 2 days after the injections.

Besides LRs, other AEs were recorded during the study. These included mainly treatment-emergent AEs (TEAEs), such as local and systemic ADRs. Systemic reactions (SRs, single symptoms according to the assessment of the investigator) and systemic adverse drug reactions (SADRs, defined as combinations of SRs) were graded according to the German AWMF classification system (Arbeitsgemeinschaft der Wissenschaftlichen Medizinischen Fachgesellschaften) [11]. In this classification, grade I reactions comprise mild systemic symptoms, grade II reactions involve moderate multisystem manifestations, grade III reactions are considered severe, and grade IV reactions are life-threatening. Although the AWMF classification is primarily used in Germany, its grading principles are broadly comparable to the internationally used WAO-based severity classifications.

All AEs were coded using MedDRA, version 25.0.

### 2.4. Definition of the Start of the Birch Pollen Season

Due to the late approval of this trial (7 March 2023), some patients received maintenance injections after the onset of the birch pollen season.

The beginning of the birch pollen season 2023 was determined individually for each study centre based on regional forecast data from the German Weather Service (DWD). The averaged threshold over all study centres was set to <2 (less than the moderate pollen count): the first day with an average expected pollen count of 2 indicated the start of the birch pollen season 2023, which occurred on 9 April 2023. For more details, please refer to Table S1.

### 2.5. Subgroup Definition and Statistical Analysis

#### 2.5.1. Definition of Subgroups

To determine the safety and tolerability of EP-088-T502 treatment during the birch pollen season, the cut-off date of 9 April 2023 was chosen (see above). For the subgroup analysis, pre- vs. pre- and co-seasonal treatment, all patients who had their last treatment visit (V5) before the start of the birch pollen season 2023 were assigned to the pre-seasonal group, and those who had their last treatment visit after the start of the birch pollen season were assigned to the pre- and co-seasonal group. Both groups were compared in terms of ADRs, use of rescue medication, and lung function following treatment at V5. As some patients attended treatment visit 4 (V4) after 9 April 2023, an additional subgroup was established, comprising patients with V4 and V5 after 9 April 2023 and patients with only V5 after 9 April 2023 (Table 1).

Since the patient collective comprised patients who received either placebo or EP-088-T502 in the previous trial T502-SIT-045, an additional subgroup analysis was performed, analysing safety and tolerability with regard to the preceding treatment. These analyses were done for all treatment visits.

#### 2.5.2. Statistical Analysis

A sample size was not calculated since this was an open, uncontrolled, non-randomised study with patients who completed the preceding study T502-SIT-045.

The statistical analyses were performed using SPSS Statistics for Windows Version 29.0.1 (Armonk, New York, NY, USA). Figures were prepared using Microsoft PowerPoint 2016 MSO (Version 2508, Build 16.0.19127.20302) 32-bit and GraphPad Prism (Version 10.1.2).

Analyses for the treatment-group subgroup were done in the Safety set; this set comprises all patients who received at least 1 injection of EP-088-T502. Analyses for the pre-seasonal and pre- and co-seasonal subgroups were done in the ITT set, since for these analyses, treatment had to be completed. The study was not prospectively designed or powered for formal comparisons between the post hoc-defined subgroups. Therefore, all subgroup analyses are considered purely descriptive in nature.

Quantitative variables were summarised with mean values, standard deviations, medians, 25–75 percentiles, and, where applicable, minimum and maximum values. Qualitative variables were described in terms of frequencies and percentages of the number of patients or events examined.

### 2.6. Ethics Statement

Prior to the start of the study, the study protocol, patient information and informed consent form, details of patient recruitment, and other relevant study documents were reviewed and approved by the Ethics Committee (EudraCT No. 2022-004082-20, study code: T502-SIT-059). The study was also approved by the competent authority (Paul-Ehrlich Institute, reference number: 5190/01) on 7 March 2023.

The study was conducted in accordance with ICH-GCP and the Declaration of Helsinki.

## 3. Results

### 3.1. General Study Data

The current study was conducted between March 2023 (first screening/treatment visit) and June 2023 (last end-of-study visit) with a mean/median overall study duration of 149.6/160 days and a mean/median treatment duration of 62.2/63 days. The last treatment visit was performed between 30 March 2023 and 27 April 2023.

#### 3.1.1. Allocation to Subgroups

**Figure 2:**
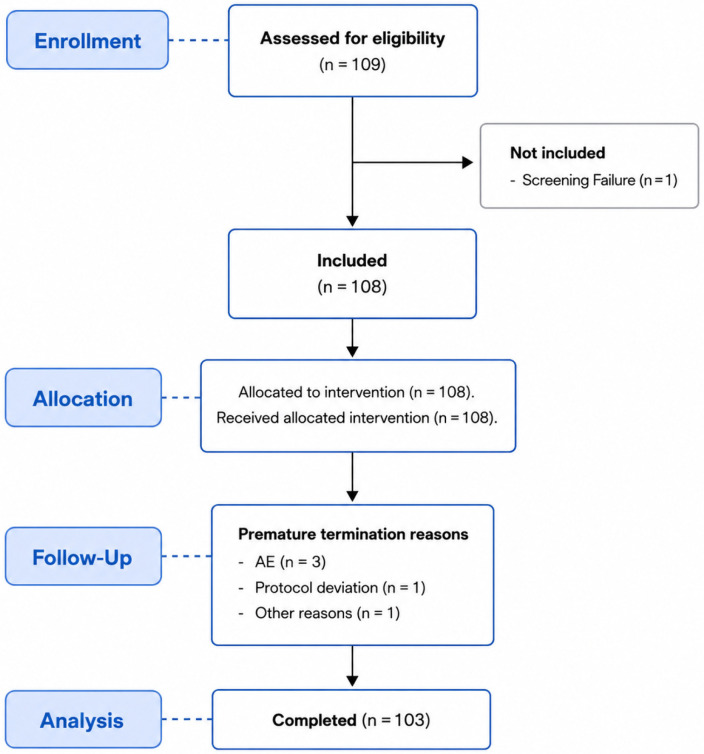
CONSORT flow chart of the study.

In total, 109 patients from the preceding study T502-SIT-045 took part in the study presented here, of whom 40 patients had belonged to the placebo group and 69 patients to the EP-088-T502 group. As one patient was deemed a screening failure, 108 out of 109 patients were included in the study and received treatment with EP-088-T502. Of the 108 treated patients, 103 (95.4%) completed treatment through V5. Overall, five patients (4.6%) dropped out: three patients due to AEs, one because of protocol deviations, and one patient due to other reasons (Figure 2). Of all patients who completed the treatment, 20 (19.4%) patients were allocated to the pre-seasonal treatment group and 83 patients (80.6%) to the pre- and co-seasonal treatment group, based on the cut-off date defined above. Of the patients in the pre- and co-seasonal group, 11 were allocated to the V4/V5-co-seasonal group and 72 to the V5-co-seasonal group.

#### 3.1.2. Exposure to EP-088-T502

Of the 103 patients who completed the treatment phase, 101 patients (93.5%) received a cumulative dose of 23,000 mTU T502. Considering the doses already received during the T502-SIT-045 trial, patients received either 23,000 mTU/mL (patients who received placebo during the T502-SIT-045 trial) or 46,000 mTU/mL EP-088-T502 (patients who received 10,000 mTU/mL during the T502-SIT-045 trial). For details on the received cumulative doses, please refer to Table S2.

### 3.2. Baseline Characteristics and General Safety Data

Demographics, baseline characteristics (including allergy-related medical history), and asthma status are shown in detail in the publication of the preceding study T502-SIT-045 by Mösges et al. [1]. In short, of all screened patients, 40 belonged to the previous placebo group and 69 to the EP-088-T502 treatment group of the 109 screened patients (108 patients were enrolled and treated); 60 were female (placebo: N = 21; EP-088-T502: N = 39) and 49 were male (placebo: N = 19; EP-088-T502: N = 30). Twenty-six patients had asthma (placebo: N = 7; EP-088-T502: N = 19), with 24 having allergic asthma (placebo: N = 7; EP-088-T502: N = 17). For details, please refer to Table S3.

No SAEs occurred during the study, and epinephrine administration was not required. No additional therapeutic or diagnostic procedures or hospitalisation were necessary due to any AE. In total, three SADRs occurred in two patients; one patient dropped out due to the SADR. All SADRs were grade I or II according to the AWMF [11]. No SADRs of grade III or IV occurred. Regarding physical examinations, vital signs, and lung function, no negative safety signals were detected irrespective of the treatment group in the preceding study.

### 3.3. Subgroup Analysis (Pre-Seasonal vs. Pre- and Co-Seasonal Treatment)

#### 3.3.1. Local Reactions

Mean/median immediate LRs (wheals) were 0.66/0.3 cm (SD: 0.81) in the pre-seasonal treatment group vs. 0.54/0.1 (SD: 0.84) in the pre- and co-seasonal treatment group. In the pre-seasonal group, the maximum wheal diameter was 2.0 cm vs. 3.5 cm in the pre- and co-seasonal treatment group. Categorisation of mean wheal diameters showed that in the pre-seasonal group, 55.0% of the wheals were of mild intensity, with 45.0% having no wheals at all. In the pre- and co-seasonal group, half of the documented wheals were of mild intensity (50.6%), while for 49.4%, no wheals developed after treatment.

**Figure 3:**
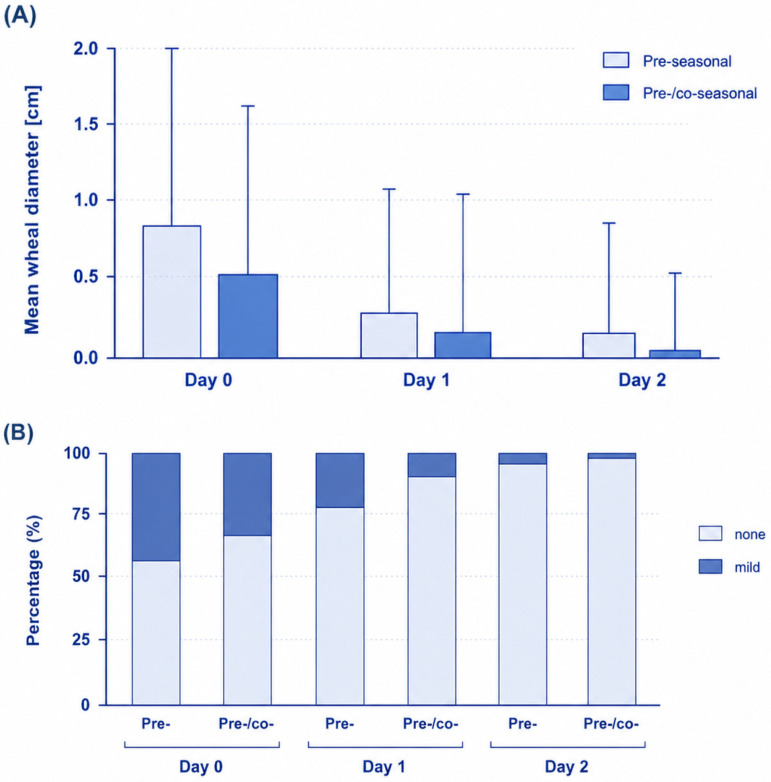
(**A**) Immediate wheal diameters at V5 in patients who were either treated pre-seasonally (pre-) or pre- and co-seasonally (pre-/co-). Data are expressed as mean + SD. (**B**) Categorical classification of late-phase wheal diameters. Data are expressed as percentage of category.

Analysis of late-phase LRs (wheals at the injection site) after visit 5 showed that mean wheal diameters were comparable to the previous visits, ranging from 0.17 to 0.94 cm in the pre-seasonal treatment group and from 0.007 to 0.60 cm in the pre- and co-seasonal treatment group. Median wheal diameters were always 0 cm, except on the day of injection in the pre-seasonal treatment group (median: 0.2 cm). Maximum wheal diameters were 4.8 cm in the pre-seasonal treatment group and 10.0 cm in the pre- and co-seasonal treatment group (Figure 3).

**Figure 4:**
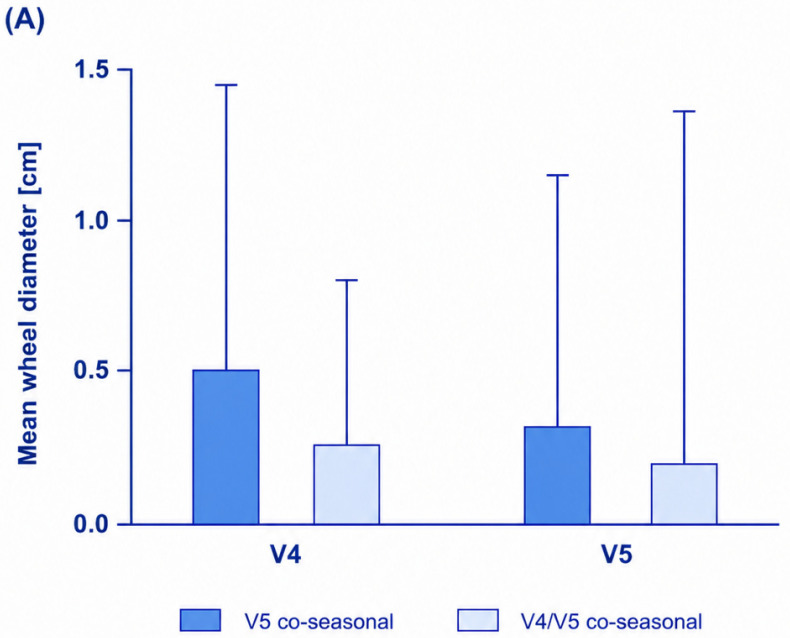

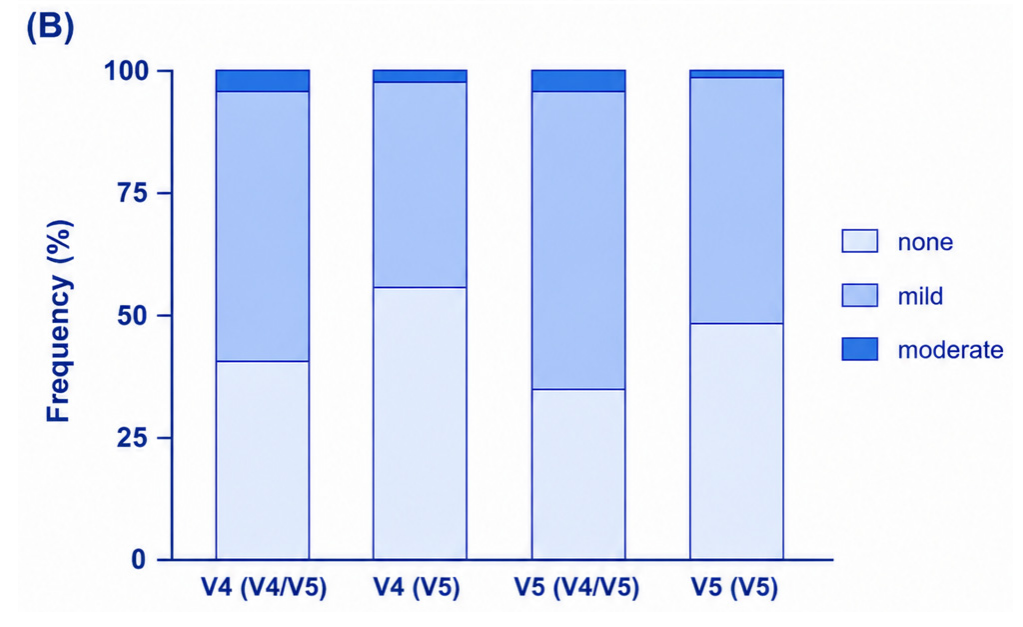
(**A**) Immediate wheal diameters at V4 and V5 in patients who were either treated co-seasonally (co-) at V4 and V5 or only at V5. Data are expressed as mean + SD. (**B**) Categorical classification of late-phase wheal diameters. Data are expressed as percentage of category.

Analysis of both co-seasonal subgroups (co-V5 and co-V4/V5) revealed that LRs were not influenced by the time point of injection (before or during the birch pollen season) at both visits: At V4, mean/median wheal diameters were 0.35/0.15 cm (SD: 0.49) in patients in the co-V4/V5 group and 0.51/0 cm (SD:0.99) in patients in the co-V5 group. At V5, mean/median wheal diameters were 0.43/0.15 (SD: 0.66) and 0.53/0 cm (SD: 0.87), respectively (Figure 4).

Late-phase LRs were documented by the patients on the day of injection (in the evening, day 0) and on days 1 and 2 following the injections. All documented late-phase LRs were of mild intensity (<5 cm) or did not occur at all. Overall, 50% of measurements showed wheals of 0 cm. Figure S1 shows the distribution of late-phase local reactions stratified according to treatment allocation in the preceding T502-SIT-045 study.

#### 3.3.2. Adverse Events

With the cut-off date of 9 April 2023, nine TEAEs were documented in five patients belonging to the pre- and co-seasonal treatment group; five TEAEs occurred in patients belonging to the V5-co-seasonal group, and four AEs occurred in patients in the V4/V5-co-seasonal group. Five TEAEs were rated as unrelated to the treatment, and four AEs were rated as possibly related to the treatment.

At V5 and following V5, 24 TEAEs occurred in total, with four in the pre-seasonal group and 20 in the pre- and co-seasonal group. With regard to the percentages of patients experiencing these events, 20% of patients in the pre-seasonal group experienced a TEAE, and 24% of patients in the pre- and co-seasonal group experienced a TEAE. Four (16.7%) of the TEAEs were rated as related to the treatment, eight (33.3%) were rated as possibly related, and 12 (50.0%) were rated as unrelated to the treatment.

One of these AEs was classified as an SADR and occurred after V5. The patient (belonging to the pre- and co-seasonal and the V5-co-seasonal group) complained of generalised itching. The SADR was rated as grade I according to the AWMF [11]. Symptoms were resolved after cetirizine administration on the following day. The patient continued the study until V7 (Table 2).

The nine TEAEs resulted in 10 MedDRA codes (PTs). Regarding LRs other than wheals and redness, one was documented as “injection-site pruritus” in a patient belonging to the pre- and co-seasonal treatment group (V5 co-seasonal group).

#### 3.3.3. Use of Rescue Medication

Following V5, for a reduction in the injection-site reactions, only two tablets of bilastine were taken by two patients, both belonging to the pre- and co-seasonal treatment group. With regard to the V5-co-seasonal and V4/V5-co-seasonal treatment groups, one tablet was used in each group. After V4, four tablets were used with three patients in the V5-co-seasonal group and one patient in the V4/V5-co-seasonal group.

#### 3.3.4. Effects on Lung Function

Comparing the lung function tests of all asthmatic patients (N = 25) before and after treatment at V5 showed that lung function was not affected by the treatment despite conducting the treatment during the birch pollen season (Table S4).

### 3.4. General Safety and Tolerability of EP-088-T502 Treatment with Regard to the Preceding Treatment Group

#### 3.4.1. Local Reactions

**Figure 5:**
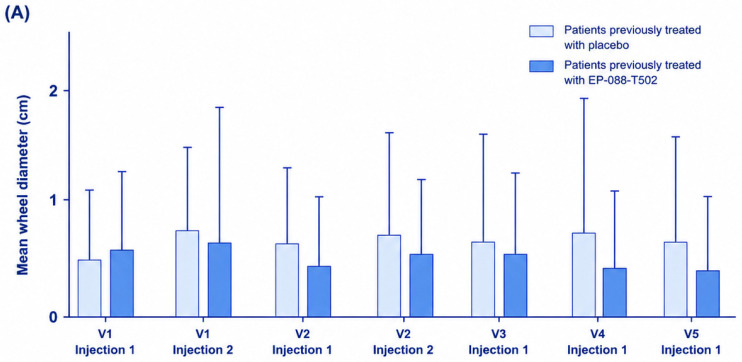

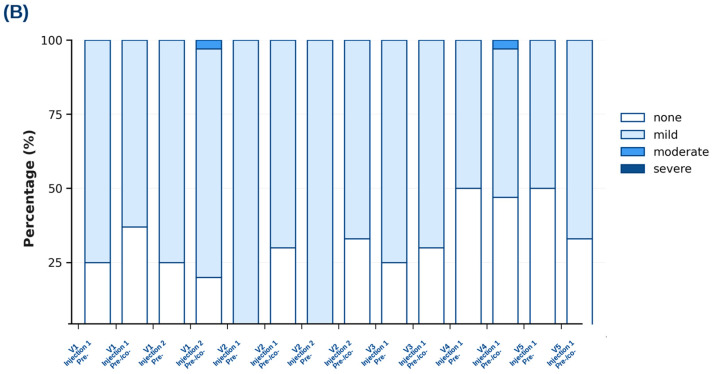
(**A**) Immediate wheal diameters of immediate local reactions, with regard to treatment in the preceding study. Data are expressed as mean + SD. (**B**) Categorical classification of late-phase wheal diameters, with regard to treatment subgroup—pre-seasonal or pre- and co-seasonal. Data are expressed as percentage of category.

Immediate LRs (wheals at the injection site occurring within 30 min after treatment) ranged from 0 to 10.0 cm over all visits. Mean/median wheal diameters ranged from 0.42/0 cm (at V4 for participants who received EP-088-T502 in the preceding study) to 0.83/0.50 cm (at V2 for participants who received placebo in the preceding study, Figure 5A). Wheal diameters were categorised into mild/moderate/severe (<5 cm/5–10 cm/>10 cm) or no wheal at all. Based on the number of patients, 32% to 53% of the patients did not experience immediate LRs during one of the treatment visits. Only one severe LR was documented throughout the treatment phase. Most of the immediate LRs were of mild intensity, independent of the previous treatment group (Figure 5B).

Late-phase LRs were of mild intensity (<10 cm) (Figure S1). Wheals with moderate intensity (10–20 cm) did not occur, while four measurements (following the first treatment visit) in patients who were previously treated with placebo were rated as severe. On average, at least 50% of the measurements documented for each day were 0 cm (no wheal, Figure S1).

#### 3.4.2. Adverse Events

Among the 108 patients who were included in the study and treated with EP-088-T502, 44 (40.7%) patients experienced 128 AEs, of which 17 (42.5% of all patients treated with placebo in the preceding study) belonged to the previous placebo group and 27 (39.1% of all patients treated with EP-088-T502 in the preceding study) to the previous EP-088-T502 group. Of all AEs, 113 were TEAEs, with 54 in the previous placebo group and 59 in the previous EP-088-T502 group. Sixty-five (57.5%) of all TEAEs were related to the treatment (previous placebo: N = 34; previous EP-088-T502: N = 31), 24 (21.2%) were rated as possibly related (previous placebo: N = 8; previous EP-088-T502: N = 16), and 24 (21.2%) were rated as unrelated to EP-088-T502 (previous placebo: N = 12; previous EP-088-T502: N = 12). Associating the (possibly) related TEAEs to the number of injections (N = 736), 8.8% of all injections led to a related TEAE, and 3.4% led to possibly related TEAEs.

Medical coding of the 113 TEAEs resulted in 123 MedDRA PTs. Regarding LRs at the injection site other than wheals and redness, the most frequently reported were PT “injection-site reaction” (N = 21, 17.1% with previous placebo: N = 6 and previous EP-088-T502: N = 15), followed by PT “injection-site pruritus” (N = 10, 8.1%, with previous placebo: N = 5 and previous EP-088-T502: N = 5) and PT “injection-site pain” (N = 8, 6.5%, with previous placebo: N = 6 and previous EP-088-T502: N = 2).

As mentioned above, a total of three SADRs (2.3% of all AEs and 2.7% of all TEAEs) occurred in two patients who had been treated with placebo in the previous study. One of the two patients who experienced SADRs belonged to the pre- and co-seasonal treatment group: the participant reported itching of the eyes and ears, rhinitis, and angioedema as late-phase reactions following visit 1. The symptoms (all SRs grade I and rated as mild) were classified as an SADR grade II, resulting in the withdrawal of the patient from the study. Another participant (belonging to the pre- and co-seasonal group) who already experienced a late-phase SADR grade I—consisting of the SRs of generalised itching and light swelling of eyes following V2—also experienced an SADR grade I of generalised itching following visit 5. This SADR was rated as mild and grade I and resolved after treatment with cetirizine tablets (Table 2).

#### 3.4.3. Use of Rescue Medication During the Treatment Phase

In total, 48 tablets of bilastine (20 mg) were taken by the patients. Most of the rescue medication was used following the first treatment visit (N = 18 tablets, with 12 in the previous placebo and N = 6 in the previous EP-088-T502 group). Based on the number of injections (total: N = 736, previous placebo: N = 252 and previous EP-088-T502: N = 484), 6.5% of all injections required the use of rescue medication. For details, please refer to Table S5.

## 4. Discussion

When considering SCIT for seasonal pollen allergies such as birch pollen allergy, care is usually taken for safety reasons to ensure that the treatment is completed before the start of the pollen season to minimise adverse reactions associated with combined natural pollen exposure and treatment-related allergen exposure. It therefore remains unclear whether treatment during the pollen season is equally well-tolerated. To further investigate this question, we analysed data from an open phase III trial in which a subgroup of patients was treated with a birch pollen SCIT during the birch pollen season 2023. The aim was to assess whether the safety and tolerability of a pre- and co-seasonal treatment differed from that observed with a purely pre-seasonal treatment regimen.

SLIT is generally considered to have a favourable safety profile for treatment during the pollen season, as the allergens are administered in the form of drops or tablets under the tongue, whereas SCIT involves subcutaneous allergen administration. The sublingual administration minimises the risk of serious side effects, such as anaphylactic reactions, associated with SCIT. Although SLIT is generally associated with a lower risk of systemic reactions due to its route of administration, direct comparisons between SLIT and SCIT should be interpreted cautiously because of fundamental differences in treatment modality, allergen exposure, and safety assessment methodologies [4]. However, pre-seasonal treatment with SCIT faces challenges, since, due to climate change, the pollen seasons are starting earlier each year [5,10]. SCIT is generally planned well in advance before the pollen season. Nevertheless, it may happen that, despite careful treatment planning, SCIT has to be carried out during the pollen season, which may increase the risk of adverse reactions. The use of SCIT products with favourable tolerability profiles may reduce this risk.

The mannan-conjugated birch pollen allergoid (EP-088-T502) is safe and well-tolerated, as demonstrated in previous studies [1,6]. However, in these studies, treatment was always completed before the start of the respective birch pollen season. In line with these studies, up-dosing (over the first two visits) and the beginning of the maintenance phase (starting with V3) were likewise performed pre-seasonally in the study presented here. Consistent with previous studies with EP-088-T502, most ADRs occurred during the up-dosing phase: herein, two SADRs comprising six symptoms (SRs) occurred in two patients during up-dosing, which is consistent with findings from previous studies, and one SADR occurred at V5 during the birch pollen season. Therefore, it remains unclear whether this reaction was attributable to treatment administration, natural pollen exposure, or a combination of both factors. From the 83 patients who had their last treatment visit during the birch pollen season, one patient (1.2%) experienced a systemic adverse drug reaction. The exact 95% confidence interval according to the Clopper–Pearson method ranged from 0.03% to 6.5%, reflecting the uncertainty associated with this rare event. These findings are consistent with previous reports with the same product [1,6,7], where the patients were treated pre-seasonally, and other studies [8,9,11,12]. However, it is challenging to compare the results presented here with other studies that have used different products (e.g., sublingual AIT) and treatment regimens.

In addition, immediate local reactions (wheals at the injection sites measured 30 min after the injection) were generally of mild intensity (<5 cm) and were more pronounced during the first treatment visits, but decreased over the course of treatment. Of note, in many patients, reactions did not occur at all. Importantly, in most of the patients (at least 50%), LRs did not develop at all (documented as 0 cm), which was the case for immediate as well as late-phase reactions. Focusing on local reactions following treatment during the birch pollen season (V4 and V5), no differences were observed in the occurrence and the severity of the wheals when compared to visits 4 and 5 conducted before the birch pollen season. Here, again, 50% of all measurements indicated no wheal at all. These findings are consistent with a favourable tolerability profile of EP-088-T502. Besides LRs like wheals, other unsolicited LRs can also occur, for example, injection-site pruritus, injection-site pain, etc. In the study presented here, these reactions were rare throughout the treatment phase. In particular, when looking at the treatment visits performed during the birch pollen season, no unsolicited LRs occurred in any of the patients. These results are in line with the documented use of rescue medication, which could be taken to alleviate symptoms due to side effects. Consistent with the reduction in adverse reactions, the use of rescue medication also decreased over the course of the study.

Allergic asthma is a common comorbidity in (birch pollen) allergic patients. Especially during the pollen season, symptoms may worsen during pollen exposure. In the previous studies conducted with EP-088-T502, no clinically relevant negative effects of the treatment on lung function of asthmatic patients were detected. However, these measurements were carried out before the birch pollen season, in the absence of pollen exposure. In the study presented here, analysis of lung function tests in asthmatic patients showed, in line with previous studies, that EP-088-T502 had no negative impact on lung function.

Since the patients who participated in the study presented here completed the preceding DBPC study T502-SIT-045, we additionally investigated whether patients previously treated with placebo might experience a higher rate of ADRs than patients who have already been treated with EP-088-T502. Notably, all SADRs occurred in patients who were previously treated with placebo. Regarding LRs (immediate and late-phase wheals at the injection site), the measured mean wheal diameters were similar in both treatment groups throughout the study. With regard to adverse events, 41% of all patients experienced at least one TEAE.

### Limitations

A major limitation of the present study is the potential selection bias resulting from the rollover design. Only 36.6% of patients from the preceding trial participated in the present follow-up study, and the majority had previously received active EP-088-T502 treatment. Consequently, the study population may have been enriched for patients who had already demonstrated good tolerability to treatment. This may have contributed to an underestimation of treatment-related adverse reactions and may limit the generalisability of the findings to broader treatment-naïve populations. The loss of numerous patients was due to some trial sites being unable to participate in the follow-up study, including one of the main recruiting sites following the unexpected death of its principal investigator. The proportion of placebo patients that was rolled over in the T502-SIT-059 study was 40.4%, whereas 34.7% of all EP-088-T502 patients from the preceding study participated in the T502-SIT-059 study. Here, the majority of patients (N = 69, 63.3%) were previously treated with EP-088-T502, representing a 1.7-fold higher proportion than that of the placebo group (N = 40, 36.7%). This affects the safety aspect of this study, since patients who tolerated the treatment in the first year of treatment are likely to tolerate the treatment in the second year. This may have enriched the cohort with patients who had already tolerated the treatment well and potentially influenced the observed safety profile.

The “cut-off” design of this study was determined post hoc based on the late approval of this study; therefore, group sizes were based on post hoc parameters and could not be controlled prospectively (e.g., through randomisation to the pre-seasonal or pre- and co-seasonal group). As a result, the (subgroup) sizes are very heterogeneous, with, e.g., 20 patients in the pre-seasonal group and 83 patients in the pre- and co-seasonal group, representing a nearly 1:4 ratio. In addition, co-seasonal treatment was not prospectively planned.

As subgroup allocation was performed post hoc and resulted in markedly unequal subgroup sizes, the study was not powered for inferential comparisons between groups. Consequently, all findings should be interpreted descriptively and considered hypothesis-generating rather than confirmatory. The wide confidence intervals around rare safety events further illustrate the limited precision of subgroup-specific risk estimates.

## 5. Conclusions

In this descriptive post hoc subgroup analysis, no apparent safety signal suggesting an increased risk associated with co-seasonal administration of EP-088-T502 was observed. The safety profile appeared descriptively similar to that observed during pre-seasonal treatment. However, due to the post hoc subgroup allocation, unequal subgroup sizes, descriptive statistical approach, and limited statistical power, these findings should be interpreted cautiously and require confirmation in adequately powered prospective studies.

## Figures and Tables

**Table 1:** Definition of subgroups according to the date of the last treatment visits (V4 and V5).

**Pre-seasonal**	--
**Pre- and co-seasonal**	Co-seasonal V5
	Co-seasonal V4 and V5

**Table 2:** Systemic reactions and evaluation according to the AWMF and cumulated systemic adverse drug reactions (SADRs).

Patient	Start of SADR	Description Symptoms	Severity of the SR	SR Grade (AWMF)	SADR Grade (AWMF)	Action Taken with IMP	Treatment of SADR
Patient 1 (previous placebo)	After V1 (late phase)	Angioedema	Mild	Grade I	Grade II	Permanently discontinued	None
		Pruritus of eyes	Mild	Grade I			
		Rhinitis	Mild	Grade I			
		Itching in inner ears	Mild	Grade I			
Patient 2 (previous placebo)	After V2 (late phase)	Generalised itching	Mild	Grade I	Grade I	Dose split	None
		Light swelling of eyes	Mild	Grade I			
	After V5 (late phase)	Generalised itching	Mild	Grade I	Grade I	Not applicable	Rescue medication

## Data Availability

Data will be available from the corresponding author upon reasonable request.

## Supplementary Materials

The following supporting information can be downloaded at https://www.mdpi.com/article/10.3390/jcm15145532/s1, Table S1. Peak pollen seasons; Table S2: Mean cumulative dose, including doses received during the T502-SIT-045 trial—S-set; Table S3: Summary of all patients; Figure S1: Local reactions in patients previously treated with placebo or EP-088-T502 in the preceding T502-SIT-045 study; Table S4: Asthma at V5; Table S5: Rescue medication use, with regard to treatment in the preceding study (tablets of bilastine 20 mg).

## Abbreviations

The following abbreviations are used in this manuscript:

ADR	Adverse drug reaction
AE	Adverse event
AIT	Allergen immunotherapy
AWMF	Arbeitsgemeinschaft der Wissenschaftlichen Medizinischen Fachgesellschaften
CSMSplus	Combined symptom and medication score plus
DBPC	Double-blind, placebo-controlled
DWD	German Weather Service (Deutscher Wetterdienst)
EP-088-T502	Mannan-conjugated birch pollen allergoid
EudraCT	European Union Drug Regulating Authorities Clinical Trials Database
GCP	Good Clinical Practice
ICH	International Council for Harmonisation
ICH-GCP	International Council for Harmonisation Good Clinical Practice
ITT	Intention-to-treat
LR	Local reaction
MCT	Microcrystalline tyrosine
MedDRA	Medical Dictionary for Regulatory Activities
MPLA	Monophosphoryl lipid A
mTU	Modified therapeutic units
PT	Preferred term
RQLQ	Rhinoconjunctivitis Quality of Life Questionnaire
SADR	Systemic adverse drug reaction
SAE	Serious adverse event
SCIT	Subcutaneous immunotherapy
SD	Standard deviation
SIT	Specific immunotherapy
SLIT	Sublingual immunotherapy
SPSS	Statistical Package for the Social Sciences
SR	Systemic reaction
TEAE	Treatment-emergent adverse event
TLR	Toll-like receptor
V1-V7	Study visits 1 to 7
