# Supplementary material for: Subcutaneous Immunotherapy with Mannan-Conjugated Birch Pollen Allergoids in a Pre- and Co-Seasonal Treatment Regimen: An Exploratory Post Hoc Subgroup Analysis of Safety and Tolerability
Source: J Clin Med. 2026 Jul 15;15(14):5532. doi: 10.3390/jcm15145532 (PMC13412818; doi:10.3390/jcm15145532)
Supplement: Supplementary file 1 [file jcm-15-05532-s001.zip › jcm-4355624-supplementary.pdf]

Table S1. Peak Pollen Seasons.

Trial Site				Start Peak	End Peak
08 Hamburg	02 Itzehoe			17.04.2023	30.04.2023
05 Bonn	01 Düsseldorf			17.04.2023	30.04.2023
14 Berlin				17.04.2023	30.04.2023
10 Saalfeld				17.04.2023	30.04.2023
03 Dresden	06 Leipzig	15 Dresden	18 Dresden	17.04.2023	30.04.2023
04 Dreieich				13.04.2023	26.04.2023
07 Heidelberg				02.04.2023	15.04.2023
13 Stuttgart				17.04.2023	30.04.2023

Table S2: Mean cumulative dose, including doses received during the T502-SIT-045 trial—S-set.

			Cumulative dose in 045	Cumulative dose in 059	Cumulative dose in 045 and 059 added up
Treatment Group assigned in T502-SIT-045	Placebo	Valid N	39	39	39
		Mean	0	20513	20513
		Standard Deviation	0	6464	6464
		95.0% Upper CL for Mean	0	22608	22608
		Minimum	0	3000	3000
		Percentile 25	0	23000	23000
		Median	0	23000	23000
		Percentile 75	0	23000	23000
		Maximum	0	23000	23000
	EP-088-T502	Valid N	69	69	69
		Mean	22957	22986	45942
		Standard Deviation	361	120	379
		95.0% Upper CL for Mean	23043	23014	46033
		Minimum	20000	22000	43000
		Percentile 25	23000	23000	46000
		Median	23000	23000	46000
		Percentile 75	23000	23000	46000
		Maximum	23000	23000	46000

Table S3: Summary of all patients.

Characteristics	Placebo*	EP-088-T502*	Total
Patients (n) (%)	39 (36.7)	69 (63.3)	108 (100)
Female (n) (%)	21 (19.3)	39 (35.7)	60 (55.0)
Male (n) (%)	19 (17.4)	30 (27.5)	48 (44.4)
Asthma (n) (%)	7 (6.5)	19 (17.6)	26 (24.1)
Allergic Asthma (n) (%)	7 (6.5)	17 (15.7)	24 (22.2)

*Allocation to the treatment group in the previous T502-SIT-045 trial.

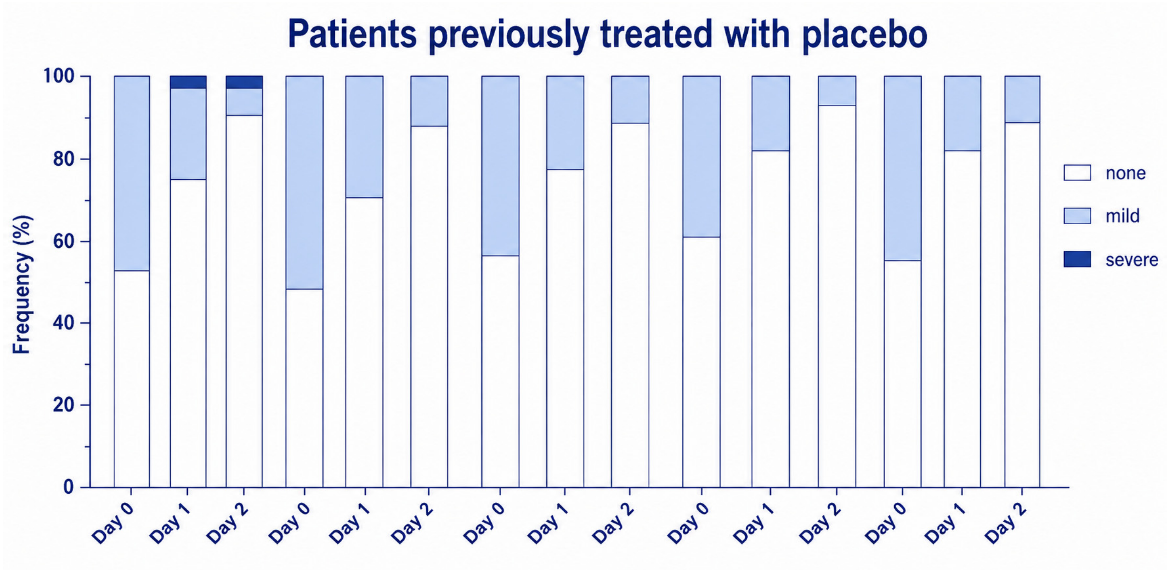

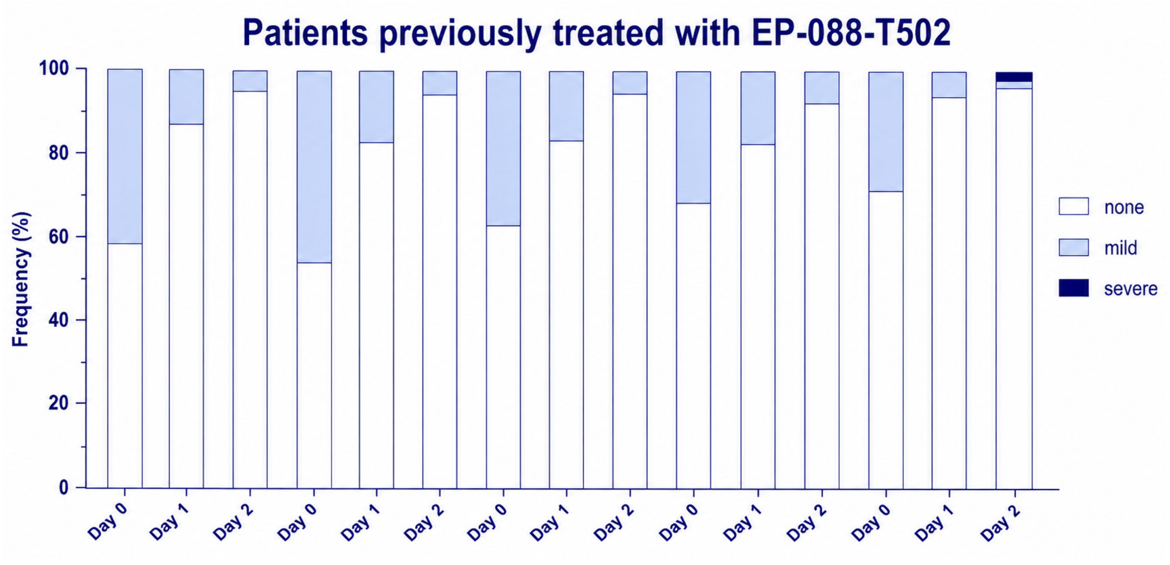

Figure S1. Local reactions in patients previously treated with placebo or EP-088-T502 in the preceding T502-SIT-045 study.

Table S4. Asthma at V5.

Subgroup		PEF Value at V5	PEF Value at V5	FEV1 measured/FEV1 predicted at V5	FEV1 measured/FEV1 predicted at V5
**Pre-seasonal**	N	4	4	1	1
	Mean	107.0750	105.8400	84.0000	80.0000
	Median	99.9700	99.5000	84.0000	80.0000
	Std.-Deviation	18.89224	17.06179		
	Minimum	93.36	93.36	84.00	80.00
	Maximum	135.00	131.00	84.00	80.00
**Pre- and co-seasonal**	N	15	15	5	5
	Mean	98.1847	97.9280	92.8000	92.0000
	Median	95.2400	96.8200	92.0000	93.0000
	Std.-Deviation	17.15435	16.89843	4.86826	3.80789
	Minimum	80.90	79.31	88.00	86.00
	Maximum	139.20	136.70	100.00	96.00

Table S5. Rescue medication use, with regard to treatment in the preceding study (tablets of bilastine 20 mg).

Visit	Previous Placebo (N=39)		Previous EP-088-T502 (N=69)		Total (N=108)	
V1	12	30.%	6	8.7%	18	16.7%
V2	7	17.9%	7	10.1%	14	13.0%
V3	2	5.1%	6	8.7%	8	7.4%
V4	1	2.6%	5	7.2%	6	5.6%
V5	2	5.1%	0	0.0%	2	1.9%
Total	24	61.5%	24	34.8%	48	44.4%

Percentages are based on the number of patients per treatment group.
